# Primary human neutrophils and monocytes migrate along endothelial cell boundaries to optimize search efficiency under static *in vitro* conditions

**DOI:** 10.1242/bio.061704

**Published:** 2025-05-13

**Authors:** Nele Honig, Christina Teubert, Lucas Lamparter, Marius N. Keller, Judith Austermann, Philipp Berger, Anne Schmitz, Christiane Rasch, Harald Nüsse, Jürgen Klingauf, Luise Erpenbeck, Johannes Roth, Milos Galic

**Affiliations:** ^1^ Institute of Medical Physics and Biophysics, Medical Faculty, University of Münster, Münster 48149, Germany; ^2^ ‘Cells in Motion’ Interfaculty Centre, University of Münster, Münster 48149, Germany; ^3^ MedK Graduate Program, Medical Faculty, University of Münster, Münster 48149, Germany; ^4^ CIM-IMPRS, International Max-Planck Graduate School, Münster 48149, Germany; ^5^ Institute of Immunology, Medical Faculty, University of Münster, Münster 48149, Germany; ^6^ Institute of Dermatology, Medical Faculty, University of Münster, Münster 48149, Germany

**Keywords:** Monocyte, Neutrophil, ER-HoxB8, HUVEC, Search optimization

## Abstract

Neutrophils and monocytes are sentinels of inflammatory signals. To reach the sites of action, both cell types attach to and then transmigrate the endothelial cell layer that lines the luminal side of blood vessels. While it has been reported that neutrophils and monocytes actively migrate along the surface of the vasculature, it remains elusive whether and how these motion patterns augment the efficiency of the immune system. Here, we conducted co-culture experiments of primary human monocytes and neutrophils, respectively, with primary human umbilical vein endothelial cells (HUVECs). Combining classical biomedical approaches with quantitative image analysis and numerical models, we find that immune cells simultaneously increase the number of sampled cells versus traveled distance and sensitivity to chemokines by migrating along endothelial cell–cell boundaries. Collectively, these findings establish search optimization of neutrophils and monocytes through limitation of motion pattern to cell–cell boundaries.

## INTRODUCTION

In response to an inflammatory signal, individual leukocytes display distinct responses. Monocytes participate in the clearance of pathogens, presentation of antigens, and aiding in tissue repair and regeneration. Complementary to monocytes, neutrophils migrate towards sites of infection, guided by chemical signals, where they neutralize pathogens. Together, their efforts contribute to protection against pathogens and the maintenance of tissue health. While individual innate immune cells vary in their responses upon exposure to chemical cues, they share the common challenge of detecting the signal and departing from the blood stream. This process, called transmigration, relies on a finely orchestrated sequence of events and requires the availability of various surface receptors. Importantly, upon attachment monocytes and neutrophils both actively migrate towards future sites of transendothelial migration (TEM) (Phillipson et al., 2006; Schenkel et al., 2004; Sumagin et al., 2010; Wojciechowski and Sarelius, 2005). In addition, monocytes were shown to patrol the luminal site of the vasculature even in the absence of inflammation (Auffray et al., 2007; Michaud et al., 2013; Wojciechowski and Sarelius, 2005). However, whether any of these motion patterns along the surface of blood vessels is spatially confined, or if this may yield any benefits, is not known.

To address this aspect, we investigated the interactions of innate immune cells with endothelial cells that form the luminal surface of the vasculature. We find both immune cell types to migrate predominantly along cell–cell boundaries of the endothelial substrate. Numerical calculations suggest that the resulting motion patter present a hitherto unknown optimization of search efficiency.

## RESULTS

### Primary human monocytes migrate along cell–cell boundaries

**Fig. 1.**
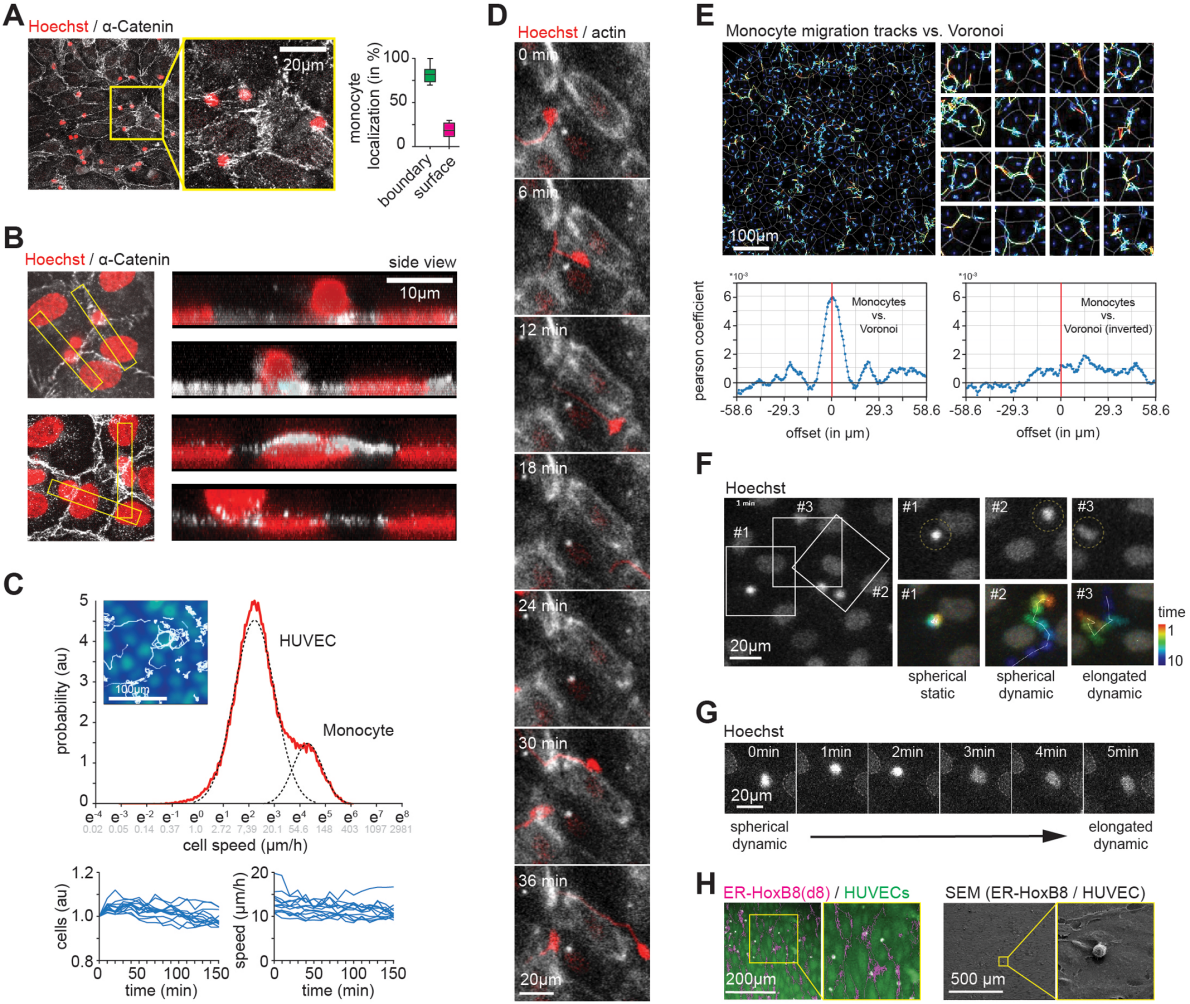
**Primary human monocytes cultured on top of monolayer of HUVECs migrate along cell–cell boundaries.** (A) Primary human monocytes (Hoechst, red) enrich at cell–cell boundaries of HUVECs (α-Catenin, white). To the right, quantification of cells located at cell–cell boundaries (green) and on top of the cell (magenta) is shown (*N*=3, *n*=196 cells). (B) Immuno-cytochemistry indicates that primary human monocytes transmigrate the HUVEC layer. Both the nuclei of monocytes (small) and HUVECs (large) are stained with Hoechst. (C) Statistical analysis of motion pattern for primary human monocytes and HUVECs. At the top, representative tracks are shown (*N*=3, *n*=12 technical repeats). The dashed lines serve as a guidance to the eye. At the bottom, to the left, graph depicting the number of cells, normalized to the initial count, throughout the entire acquisition period. To the right, graph tracking speed over time. Each blue line represents a technical repeat. (D) Primary human monocytes (Hoechst, red) migrate along cell–cell boundaries (actin, white) of HUVECs. Only the nucleus of monocytes is labelled by Hoechst. (E) Motion tracks of human monocytes on top of HUVECs. At the top, Voronoi of endothelial layer (gray) as well as tracks of monocytes and of HUVECs are shown. Insets to the right are 70 µm×70 µm. Below, to the left, cross-correlation analysis shows enrichment of monocyte tracks along endothelial cell–cell boundaries identified by Vornoi tesselation. As negative control, to the bottom right, one channel was rotated by 180° prior to cross-correlation analysis. (F) Time-lapse of primary human monocytes (red) show different migration pattern. Both, the nuclei of monocytes (small) and HUVECs (large) are stained with Hoechst. (G) Primary human monocytes change fluorescence intensity of nucleus during transmigration. Both, the nuclei of monocytes (small) and HUVECs (large) are stained with Hoechst. (H) ER-HoxB8 derived monocytes/macrophages migrate along HUVEC boundaries. To the left, HUVECs (green) and tracks of ER-HoxB8 derived monocytes/macrophages (magenta) are shown. To the right, scanning electron microscope show ER-HoxB8 derived monocytes/macrophages at cell–cell boundaries of a confluent HUVEC sheet. Scale bars: (A,D,F,G) 20 µm, (B) 10 µm, (C,E) 100 µm, (H) 200 µm and 500 µm.

In a first set of experiments, we aimed to probe localization of primary human monocytes (Fig. S1a,b) on a confluent layer of primary human umbilical vein endothelial cells (HUVECs). To that end, samples were fixed and stained with the nuclear marker Hoechst and antibodies directed against α-Catenin, which links VE-Cadherin to the actin cytoskeleton (Herrenknecht et al., 1991). Consistent with previous reports (Jakubzick et al., 2013; Wojciechowski and Sarelius, 2005) only a subfraction of monocytes did adhere sufficiently strongly to the endothelial sheet to withstand washing steps associated with fixation and staining. Localization analysis of these immune cells showed an enrichment at cell–cell boundaries of the confluent HUVEC layer (Fig. 1A).

TEM is a multi-step progress, whereby cells tether, roll and adhere to endothelial cells, followed by transmigration from the luminal side of the blood vessel into the tissue (Butcher, 1991; Springer, 1994). To determine which of these steps were present in our system, we acquired 3D confocal sections of our co-culture system (Fig. 1B). We found monocytes on top of a confluent HUVEC sheet, cells that were fixed while transmigrating through a junctional gap in the endothelial sheet, as well as cells located underneath the confluent HUVEC layer. Notably, the nucleus of monocytes located on top of the layer appeared spherical, while the nucleus of cells underneath the endothelial sheet was flattened along the z-axis (Fig. 1B, right panels). While monocyte extravasation at non-inflamed sites is less commonly observed, these findings are consistent with previous reports (Schenkel et al., 2004).

The experiments to this point show successful TEM of primary human monocytes in our model system. The data, however, does not explain whether the high prevalence of monocytes at cell–cell boundaries is reflective of different stages of TEM at endothelial cell–cell boundaries or migration along these structures prior to TEM. To address this question, we probed in a second set of experiments the cell dynamics of both cell types, using an acquisition rate of 1 frame per minute (Movie 1). Consistent with differences in migration speed between immune and endothelial cells, analysis of displacement over time showed two distinct populations (Fig. 1C, top). For all biological and technical repeats, we monitored median cell density (Fig. 1C, bottom left) and cell speed (Fig. 1C, bottom right) over time. No significant changes were observed for either parameter, arguing that the system remained stable throughout the acquisition window. To further validate the stability of the system, we checked for the spontaneous occurrences of cell death. Across all movies, we observed only a few events, further consolidating the overall good health of the co-culture system over the whole acquisition window. In the rare occasions, where dissociation of the nucleus was observed (i.e. apoptosis), we find recruitment of monocytes to dying cells, followed by local swarming and an uptake of cell debris (Movie 2). Curiously, monocytes did not migrate in a straight line, as a chemokine gradient would dictate, but seemed to follow an indirect path to the source. Using co-culture system where immune and endothelial cells were differentially labelled, we find that primary human monocytes selectively migrate along endothelial cell–cell boundaries (Fig. 1D; Movie 3). To quantify this observation, while minimizing unwanted effects on binding affinity or migration due to overexpression or fluorescence labelling, we in a next set of experiments used the Voronoi derived from the nuclear positions as proxy of endothelial boundaries. Comparison of Voronoi tessellation versus VE-Cadherin in fixed cells labelling confirmed the validity of this approach (Fig. S1C). To probe whether the nucleus is a valid reference point to track immune cells, we then compared the coordinates of the center of mass determined via cell shape versus nucleus (Fig. S1D). The median distance between these two points was below 2 µm, which is negligible at the time and length scales relevant in this study. Consistent with our previous results, we find selective migration of primary human monocytes along endothelial cell–cell boundaries (Fig. 1E).

A morpho-kinetic analysis of monocytes identified three distinct types of migration (Fig. 1F and Movie 4): Tracks that displayed no active migration, but moved at the same pace and in the same direction as the underlying HUVEC layer. Tracks that spent extended periods of time at one site and then actively migrated to the next spot. And tracks that rapidly migrated without extended dwell times at a particular site. While the former two groups were associated with round/bright nuclear staining, the latter one was associated with a dim/meandering nuclear staining (Movie 5). Again, we find mutual spatial exclusion of the nucleus of monocytes and HUVECs for all three motion types, indicative of an enrichment of monocytes at cell–cell boundaries before, during and after TEM. We further find the nuclear staining to transition within less than one minute from bright/circular to dim/meandering shape (Fig. 1G and Movie 6). Unlike the transition from bright to dim, we did not observe any reverse transitions.

Collectively, the data to this point establish dynamic migration of monocytes along the surface of the vasculature. To further consolidate these observations, we took advantage of ER-HoxB8 cells that display a monocyte/macrophage-like phenotype upon induction (Wang et al., 2006) (Fig. S2A,B). Much like with primary human monocytes, we find in fixed samples enrichment of ER-HoxB8 derived monocytes/macrophages at cell–cell junctions of HUVECs, as well as preferential migration along these boundaries (Fig. 1H and Fig. S2C).

### Neutrophils also migrate along cell–cell boundaries

Having established that monocytes preferentially migrate along cell–cell boundaries, we next aimed to probe the motion pattern of another innate immune cell type. Published work reported intraluminal crawling of neutrophils to distant emigration sites in wild-type mice (Phillipson et al., 2006). If this migration is restricted in any particular fashion, however, has remained elusive.

**Fig. 2.**
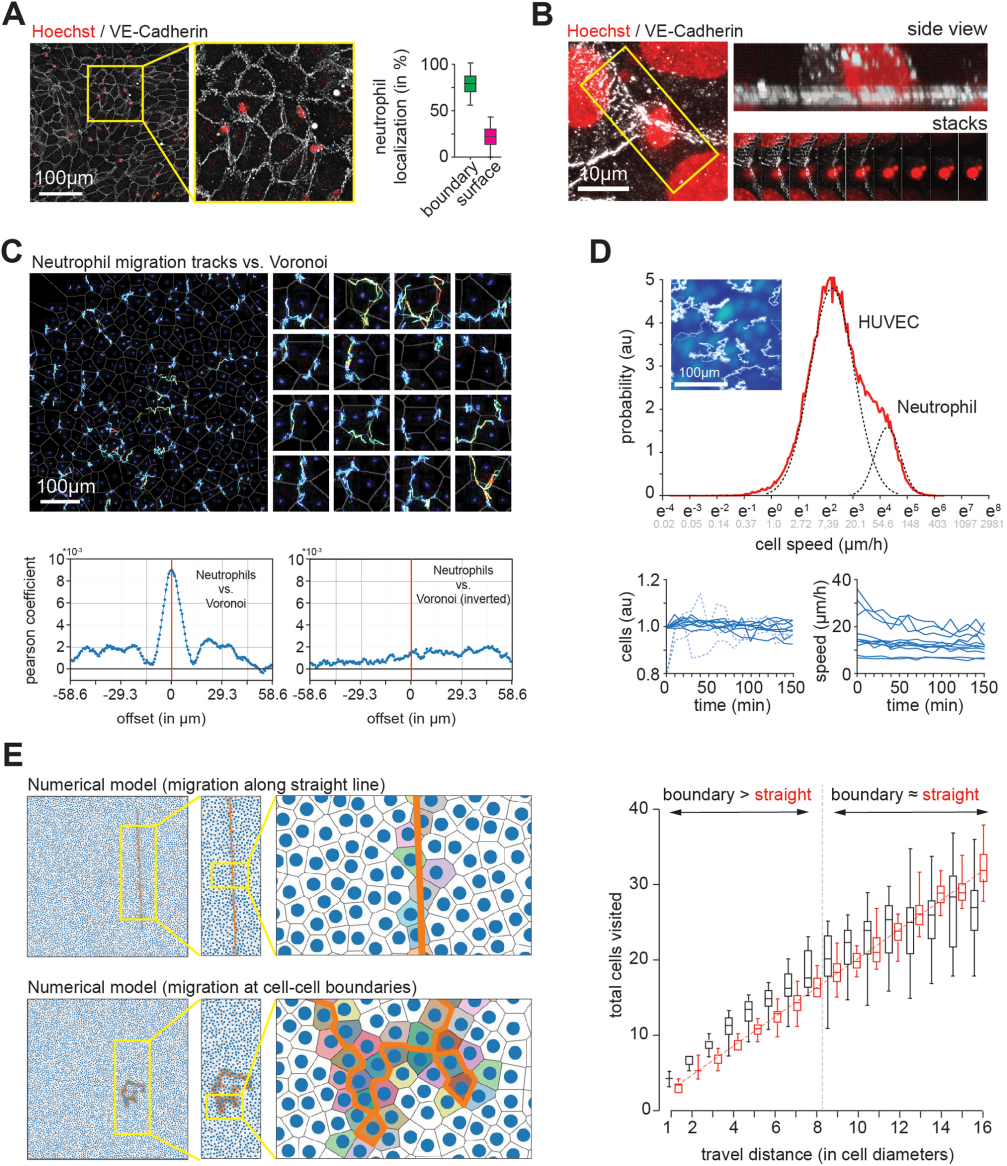
**Primary human neutrophils migrate along cell–cell boundaries of HUVECs.** (A) Primary human neutrophils cells (Hoechst, red) enrich at cell–cell boundaries of HUVECs (VE-Cadherin, white). To the right, quantification of neutrophils located at cell–cell boundaries (green) and on top of the cell (magenta) are shown (*N*=3, *n*=185 cells). (B) Immuno-cytochemistry indicates that primary human neutrophils cells migrate on top of HUVECs. Both, the nuclei of monocytes (small) and HUVECs (large) are stained with Hoechst. (C) Motion tracks of human neutrophils cells on top of HUVECs. At the top, Voronoi of endothelial layer (gray) as well as tracks of neutrophils and of HUVECs are shown. Below, to the left, cross-correlation analysis shows enrichment of neutrophil tracks along endothelial cell–cell boundaries identified by Vornoi. As negative control, to the bottom right, one channel was rotated by 180° prior to cross-correlation analysis. (D) Statistical analysis of motion pattern of primary human neutrophils and HUVECs. At the top, representative tracks are shown (*N*=3, *n*=12 technical repeats). The dashed lines serve as a guidance to the eye. At the bottom, to the left, number of cells, normalized to the initial count, throughout the entire acquisition period. To the right, speed over time. Each blue line represents a technical repeat. (E) Numerical model indicates that migration along cell–cell boundaries posits a better search strategy than a straight line for short distances. To the left, representative runs along straight line (top) and along cell–cell boundaries (bottom) are shown. To the right, a quantification of sampled endothelial cells versus walking distances is shown for both motion types. Scale bars: (A,C,D) 100 µm, (B) 10 µm.

As above, we in a first set of experiments co-cultured primary human neutrophils (Fig. S3A,B) with HUVECs, and then fixed and stained the sample with markers directed against the nucleus and VE-Cadherin. Similar to monocytes, we find significant enrichment of primary human neutrophils at HUVEC cell–cell boundaries (Fig. 2A), with the immune cells located on top of the layer (Fig. 2B). Next, taking again advantage of the Voronoi tessellation, we probed neutrophil motion pattern. We find that neutrophils migrated, as monocytes, selectively along cell–cell boundaries (Fig. 2C). Finally, we quantified cell speed (Movie 7). As before, we find a bimodal distribution of migration speed (Fig. 2D), indicative of rapid neutrophil motion on top of a slowly moving HUVECs layer. No significant changes in cell number (Fig. 2D, bottom left) were observed, suggestive of a good overall health of the experimental system.

### Migration along cell–cell boundaries improves the search efficiency

Migration patterns were reported to influence the search efficiency of single cells (Begemann et al., 2019). To address the question how migration along cell–cell boundaries may affect the search ability of immune cells, we derived a reductionist model that reconstitutes the basic motion pattern observed for neutrophils and monocytes, respectively, on top of the vasculature. We then let individual immune cells to migrate for a defined number of endothelial cell diameters, and scored the number of endothelial cells that were touched by the immune cell. For track lengths of less than eight cell diameters, migration along the cell–cell boundaries allowed sampling more endothelial cells than a straight line (Fig. 2E; Fig. S4 and Movie 8). For longer tracks, a straight line becomes more efficient in our numerical model. These findings are relevant, as they suggest that at physiologically relevant length-scales restricting the motion of monocytes to the cell–cell boundaries outperforms motion along a straight line, which is generally considered to be the optimal search strategy for planar system.

## DISCUSSION

Monocytes were described to actively move from sites of adhesion to the nearest junction for TEM (Schenkel et al., 2004). However, a large fraction of monocytes will migrate multiple cell diameters along the luminal site of endothelial cells *in vitro* (Schenkel et al., 2004) and *in vivo* (Sumagin et al., 2010). Similarly, neutrophils will not pick the nearest available junction, but migrate along the vasculature to hotspots from which they depart into the tissue (Grönloh et al., 2023). Whether these ‘long walks’ along the endothelial layer are spatially restricted in a particular manner, or if such a bias may yield any benefits, has to date not been assessed. Here, we probed the motion pattern of primary human neutrophils and monocytes on an endothelial layer. We find that monocytes and neutrophils, respectively, enrich at cell–cell boundaries of HUVECs, and dynamically move along these structures.

Considering that the presented work was conducted *in vitro*, several limitations apply. First, since the experiments lack fluid shear forces, it remains elusive to what degree strength and flow-pattern of the blood stream may affect the ability of immune cells to attach and migrate. Second, blood vessels differ biophysically from the microfluidics chamber used in this study. It is thus plausible to envision that difference in substrate stiffness and diameter of the endothelial tube may affect adhesion and transmigration of immune cells. Third, while suitable for long-term co-culturing experiments, the medium does not fully reconstitute the blood serum that differs not only in composition but also in viscosity, likely further modifying the immune-endothelial interactions through paracrine signaling. Despite these limitations, reports of immune cells migrating both with and against the bloodstream *in vivo* (Auffray et al., 2007) argue that the observed patrolling function along the endothelial surface is also relevant in living systems*.*

Conceptually, these findings yield two major conclusions. First, motion along cell–cell boundaries increases search efficiency. Neutrophils and monocytes not only respond to diffusive, but also to membrane-bound signals. For instance, non-classical monocytes were described to sample the luminal site of the vasculature for unhealthy particles (Auffray et al., 2007; Michaud et al., 2013). It is thus plausible to envision that some of the motion pattern observed at the luminal side of the vasculature may reflect patrolling of non-classical monocytes. Similarly, neutrophils transmigration has been described to occur at hotspots (Grönloh et al., 2023). While fundamentally different objectives, both tasks will benefit by increasing the number of sampled endothelial cells per traveled distance. Second, given that on the luminal side of the endothelial layer the concentrations of soluble signaling cues from nearby inflamed tissue are highest at cell–cell junctions, the movement of immune cells along these boundaries is likely to augment its fidelity. Hence, to reliably detect a potential inflammation, it is beneficial to migrate along cell–cell boundaries.

Collectively, these findings establish that neutrophils and monocytes selective migrate along endothelial cell–cell boundaries to increase its search efficiency. The reason why immune cells migrate along endothelial cell–cell boundaries, however, remains elusive. Consistent with published work (Angom et al., 2022; Hillebrand et al., 2008; Oberleithner et al., 2003), we find that the nucleus of endothelial cells is strongly flattened (Fig. 1B), thus excluding topological constrains as main reason. Noteworthy, intercellular adhesion proteins were reported to play a role in the migration of monocytes (Schenkel et al., 2004) and neutrophils (Grönloh et al., 2023). Future work will determine the identity of these heterotypic cell–cell interaction sites and its relevance for immune cell function in the normal and inflamed tissue.

## MATERIALS AND METHODS

### Handling of human umbilical vein endothelial cells (HUVECs)

Commercially purchased HUVECs (Promocell, C-12208) were thawed, centrifuged at 400× ***g*** and suspended in HUVEC Medium. HUVEC Medium consists of endothelial cell growth medium 2 (EGM2) (Promocell, C22011) and Medium 199 (Earle's Salts, gibco, 31120022) with a ratio 1:1 and supplemented with 5% fetal bovine serum (FBS) (Sigma Aldrich, S0615), 50 ng/µl amphotericin B (Gibco, 15290-018), 50 µg/ml gentamicin (Gibco, 15710-049), and 0.5 U/ml heparin (Sigma Aldrich, H3149). HUVECs were cultured in 6 cm and 10 cm CellBIND surface dishes (Corning, 3295 and 3296). HUVECs were no longer used after 2-3 passages. For passaging, cells were washed with 2 ml phosphate-buffered saline (PBS) (Gibco, 10010015) and detached by incubating with 1 ml trypsin/EDTA (0.04% (w/v)/ 0.03% (w/v), Promocell, C-41010) for 3 min at 37°C. Cells were resuspended with 9 ml of culture medium and centrifuged at 400× ***g*** for 4 min. The supernatant was discarded and cells were resuspended in HUVEC medium. The cells were then seeded into a new dish. Cells were passaged 1:3, each passage after 3 days. After two passages, once the cells had reached 90-100% confluency, cells were frozen at −140°C in cryovials (Thermo Scientific, 144661), containing 90% FBS and 10% dimethyl sulfoxide (DMSO). Three days after thawing the cells, they are seeded into ibidi µ-Slide VI that are coated with collagen. About 50,000 cells were seeded into each channel.

### Isolation and culturing of primary human monocytes

Human monocytes were isolated form thrombocones that were cut open at the bottom end and put in the opening of a T75-bottle. The top end of the thrombocones was also cut open and the blood was added to the T75 bottle, which was then filled up to 120 ml with HBSS. The mixture of blood and HBSS was then mixed by swaying the bottle. 30 ml of the mixture was then layered on 20 ml of Pancoll (Pan-Biotech, density of 1.070 g/ml). Following, cells were centrifugated at 540× ***g*** for 35 min at room temperature. In the meantime, a second gradient was prepared by adding 15 ml of HBSS, 13.5 ml of Percoll (General Electric Company, density of 1.064 g/ml), and 1.5 ml of MEM 10x to centrifuge tubes. This mixture was centrifuged at 15,455× ***g*** for 12 min, and then left in the fridge. After centrifugation of the HBSS-blood-Pancoll solution, a layer of monocytes formed in the center, which was carefully removed and added to a 50 ml falcon. The falcon was then filled with HBSS and centrifuged for 10 min at 300× ***g***. The supernatant was removed and the pellet was resuspended in 1 ml of HBSS and then filled up to 50 ml with HBSS. This solution was then subjected to two more rounds of centrifugation at 170× ***g*** and 300× ***g*** for 10 min each. After removing the supernatant and resuspending the pellet with 1 ml of HBSS, 1 ml was added to the tube containing the prepared second gradient. This tube was then centrifuged at 540× ***g*** for 60 min. Afterwards, two layers should have formed. The top layer was carefully added to a new 50 ml falcon, which was then filled up to 50 ml with HBSS. This falcon was then centrifuged and filled three more times at 300× ***g***, 170× ***g*** and 300× ***g*** for 10 min each. The last pellet was resuspended with RPMI 1640 (Pan Biotech P04-17525), containing 15% FCS (Biowest, S00GF1000J), 1% Penicillin-Streptomycin (10,000 U/ml Pen, 10 mg/ml Strep, Pan Biotech, P06-07100), 1% L-Glutamine (200 mM, Pan Biotech, P04-80100), and 1% non-essential amino acids. Considering that monocytes are larger than lymphocytes, detected by forward scatter (FSC), and display lower granularity than granulocytes, detected by side scatter (SSC), the purity of monocyte populations was validated using FACS by plotting FSC vs SSC (Fig. S1A). Samples were used when purity >80%.

### Culturing and differentiation of ER-HoxB8 derived monocytes/macrophages

As previously published (Wang et al., 2006), ER-HoxB8 cells were cultured in RPMI (Promocell) containing 10% FCS and 1% Pen-Strep. The base medium was supplemented with 2 µg recombinant granulocyte–macrophage colony–stimulating factor (GM–CSF) (Immunotools), and 1 µm β–Estradiol (Sigma–Aldrich). Cells were passaged every other day in six-well chambers. For differentiation, ER-HoxB8 cells were transferred into a 15 ml centrifuge tube and washed by centrifuging at 300× ***g*** for seven minutes and resuspending in PBS. The falcon was then filled up to 12 ml with PBS and the process repeated twice. Finally, the cells were resuspended in 1 ml of the base medium, supplemented with GM-SCF but no estradiol. This counts as day 0 of differentiation.

### Isolation and culturing of primary human neutrophils

All experiments with human neutrophils were approved by the Ethics Committee of the University of Münster (Application #2021-657-f-S). Donors were fully informed about any possible risks and their right to withdrawal from the study at any time with informed consent confirmed in writing. Blood was drawn into S-Monovettes EDTA (7.5 ml, Sarstedt) and neutrophils were isolated from blood using the EasySep^TM^ direct human neutrophils isolation kit (Stemcell Technologies, 19666). They were then centrifuged at 400× ***g*** for 10 min at room temperature, resuspended in RPMI medium and counted. To determine neutrophil purity, a total of 150,000 cells were suspended in 100 µl of PBS with 15% BSA and then transferred onto a microscopy slide using a Cellspin I cytocentrifuge (Tharmac) at 500 rpm for 3 min. The cells were fixed and stained using the Panoptical Fast Staining Kit (Carl Roth, 6487). Stained cells were imaged with an Olympus BX63 microscope and analysed using cellSense Dimension 1.8 software (Olympus). Cells were counted in eight images, and the purity of neutrophils was calculated as the percentage of neutrophils relative to all leukocytes, while the erythrocyte count was determined separately (Fig. S3A,B). Samples were used when purity >90%.

### Live cell imaging

Monocytes and neutrophils, respectively, were stained with Hoechst 34580 (Themo Fisher H21486, 1:200) and CellTracker Red (Invitrogen CV34552, 1:1000) or CellMask Orange Actin (Themo Fisher A57244, 1:1000), respectively, and incubated in the dark at room temperature for 40 min. Neutrophils were centrifuged at 400× ***g*** for 10 min, while monocytes were centrifuged at 300× ***g*** for 8 min and resuspended in HUVEC medium and centrifuged again to wash out the staining. The pellet was then resuspended with 200 µl of HUVEC medium per slide. The cells in the suspension were then counted and the suspension diluted to 1.5×10^6^ cells/ml. To seed neutrophils and monocytes, respectively, 30 µl of the cell suspension was added to the channel on one side and the HUVEC medium was removed by suction. This results in 45,000 cells in each channel of the ibidi µ-slide. The slide was left to rest for 1 h at 37°C before channels were filled up with HUVEC medium and imaged.

### Immunocytochemistry

For antibody staining, neutrophils and monocytes, respectively, were seeded on HUVECs as described previously, skipping the staining with Hoechst and cell tracker. Instead, slides were washed with PBS++ and fixed with PFA 4% dissolved in PBS++. Specifically, 30 µl of PFA was first added to one side of the channel, and removed from the other side. Then, 100 µl were added to each channel. PFA was left in the channels to incubate for 10 min at room temperature. The channels were then washed three times with PBS++. Following, fixed cells were washed with PBS++, and then permeabilized. The permeabilization solution was left in the channels to incubate for 10 min at room temperature. The slides were then washed with PBS++. For blocking, 100 µl blocking solution was added to each channel and left to incubate on a shaker for 60 min at room temperature. Primary antibodies were diluted in PBST in a concentration of 1:200 and left to incubate overnight at 4°C on a shaker. Following primary antibodies were used: CD14 Monoclonal Antibody (Thermo Fisher, 60253-1-IG), VE-Cadherin Monoclonal Antibody (R&D Systems, MAB9381), α-Catenin (Atlas Antibodies, HPA063535). Secondary antibodies were diluted at a concentration of 1:1000 in PBST, and incubated in the dark at room temperature on a shaker for 60 min. Following secondary antibodies were used: Alexa fluor 488 goat-anti-mouse (Life Technologies, A11001), Alexa fluor 568 goat-anti-rabbit (Life Technologies, A11011). In some experiments, cells were alternatively stained with Hoechst (1:500) or Phalloidin (1:1000). Finally, channels were washed with PBS++. About three drops of ibidi mounting medium (ibidi, 50001) was added to the channels.

### Fluorescence microscopy

Cell migration was imaged using an inverted confocal microscope (Nikon, Eclipse Ts2) with a digital camera (Nikon DS-Fi2), using a 20× objective at 37°C and 5% CO_2_ with a frame interval of 60 s. Using multi-frame imaging allowed the recording of multiple positions per channel (i.e. technical repeats). Fixed samples, which were stained using antibodies, were imaged using Leica fluorescence microscope. Z-stacks were taken using a 63× objective. The distance between the single images in the z-stack was set to 0.5 µm.

### Scanning electron microscopy

Samples were washed three times in the chamber with PBS++, then fixed overnight using PFA (2%) and GA (2.5%) in 0.1 M sodium cacodylate buffer. Next, the samples were washed three times for 5-10 min each in PBS++. The chamber bottom was then cut out to dimensions of approximately 3.8×10 mm. Subsequently, the samples were fixed for 1 h with 1% OsO4 in PBS (without Ca and Mg). Following, an ascending alcohol series was applied (30%, 50%, 70%, 90%, followed by two rounds of 100% ethanol for 10-15 min each), followed by critical point drying using a Leica CPD300 automatic system. Finally, samples were mounted using Carbon Adhesive Tabs (Plano G3347) and sputter-coated with 15 nm of Au. Imaging was conducted with a scanning electron microscope FEI Quanta 600F at 15 kV.

### Image analysis

For tracking, movies were first pre-processed in Fiji/ImageJ (Schindelin et al., 2012) as follows: first contrast enhanced, using 0.35% saturated pixel (equalize histogram, process all), followed by image alignment via SIFT (Lowe, 2004), using following parameter conditions: initial gaussian blur: 1.6 pix, 3 steps per octave, minimum image size: 64 pix, maximum image size: 1024 pix, feature description size: 4, feature descriptor orientation bins 8, closest/next ratio: 0.92, max alignment error: 25 pix, inlier ratio: 0.05, expected transformation: translation, output: interpolate. Next, individual nuclei were identified using Stardist (Schmidt et al., 2018) with following parameters: Model: versatile (fluorescent nuclei); normalized image; percentile low: 1%, percentile high: 100%, probability/score threshold: 0.40, overlap threshold: 0.40, output type: both, ROI position: Automatic. The output was analyzed with Trackmate (Tinevez et al., 2017), using the following parameters: LoG detector, object diameter: 25, quality threshold: 0.03, simple LAP tracker, linking distance: 3, gap-closing distance: 3, gap-closing max frame gap: 30. Tracks were displayed backwards in time for 30 frames.

### Use of artificial intelligence tools

A convolutional neural network (CNN) was trained to identify morphological changes in ER-Hoxb8-derived monocytes/macrophages (Fig. S2A,B). In brief, ER-HoxB8 cells were fixed on consecutive days after differentiation, and stained with phalloidin (green) and Hoechst (red). Next, images of individual cells were circularly cropped using nuclear staining as a reference. A total of 300 RGB images from day 1 (immature ER-HoxB8 cells) and day 10 (mature ER-HoxB8 cells derived monocytes/macrophages) were used to train and validate the network, with 10 steps per epoch and a total of five epochs. Finally, the trained CNN was applied to categorize images from the individual days post. CNN was inspired by online tutorial (https://www.youtube.com/watch?v=uqomO_BZ44g). Large Language Models (LLM), namely perplexity.ai and deepseek.com, were used together with other approaches to conduct online literature search and to assist with spellchecking of self-written text.

### Numerical model

To investigate the influence of migration patterns on the search efficiency of immune cells, a computational model implemented in Python was developed (https://github.com/Die-Nase). The code models immune cell migration along cell–cell boundaries and as a straight line, thus allowing the analysis of migration strategies. The user can customize parameters such as cell radius, number of simulated runs, length of the migration path, and visualization options. Upon execution, the code generates images as JPEG files of the migration paths and provides a statistical analysis using the Mann–Whitney *U*-test to compare the distribution of visited regions between simulated migration paths at cell–cell boundaries and as straight lines. The results were visualized using box plots and saved with the generated JPEGs of the migration pathways in a separate folder.

### Statistics

Unless stated otherwise, a non-parametric rank-sum test was used to determine significance (ns for *P*>0.05; * for *P*<0.05; ** for *P*<0.01; *** for *P*<0.001). Box plots present the median, extending from the 25th to the 75th percentile. Whiskers depict to the smallest and largest values.

## Supplementary Material

10.1242/biolopen.061704_sup1 Supplementary information
