# Supplementary material for: Primary human neutrophils and monocytes migrate along endothelial cell boundaries to optimize search efficiency under static in vitro conditions
Source: Biol Open. 2025 May 13;14(5):bio061704. doi: 10.1242/bio.061704 (PMC12091226; doi:10.1242/bio.061704)
Supplement: Supplementary information [file biolopen-14-061704-s1.pdf]

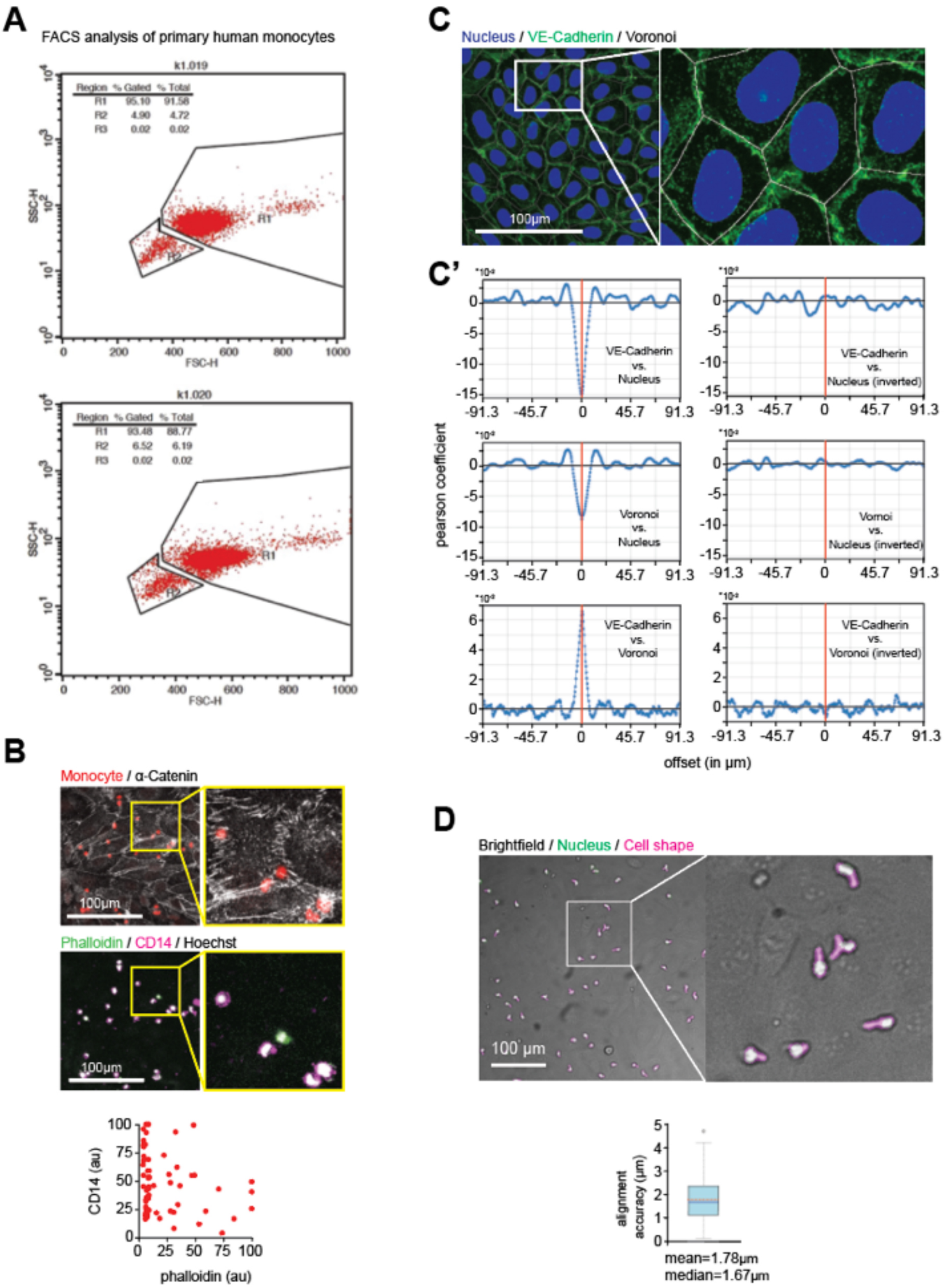

Biology Open • Supplementary information

Fig. S1. Validation of primary human monocytes co-cultured with HUVECs. (A) FACS analysis of isolated monocytes. Human monocytes were recovered from leukoreduction system chambers, purified by Ficoll-Paque (Pharmacia, Freiburg, Germany) and subsequently density gradient centrifugated. Purity of monocytes was determined by flow cytometric analysis using forward/sideward scatter. **(B)** Immuno-cytochemistry suggests presence of classical and non-classical human monocytes on top of HUVEC layer. Cells were co-cultured, subsequently fixed and stained with markers for actin (green), Hoechst (blue) and CD14 (magenta). Note cells with low CD14 and high Phalloidin levels. **(C)** Voronoi (white) derived from HUVEC nucleus (blue) aligns well with VE-Cadherin signal (green). (c') Cross-correlations analysis confirms spatial alignment of VE-Cadherin and Voronoi. Top panels show anti-correlation for VE-Cadherin vs. nucleus (left) as well as for Voronoi vs. nucleus (middle), while VE-Cadherin vs. Voronoi shows a positive correlation (right). As a negative control, in all experiments we rotated one of the channels by 180 degrees and repeated the analysis. For all conditions, this eliminated any correlation (bottom graphs), excluding the possibility that the observed peaks are random noise. **(D)** Measurement of alignment accuracy shows only a minute differences between the center of mass determined by cell nucleus (green) vs. cell shape (magenta). To the top, brightfield image of monocytes cultured on top of a confluent HUVEC layer. Below, analysis of spatial distance between the two centers of mass (n=85 cells). Scale bars, (B, C, D) 100 µm.

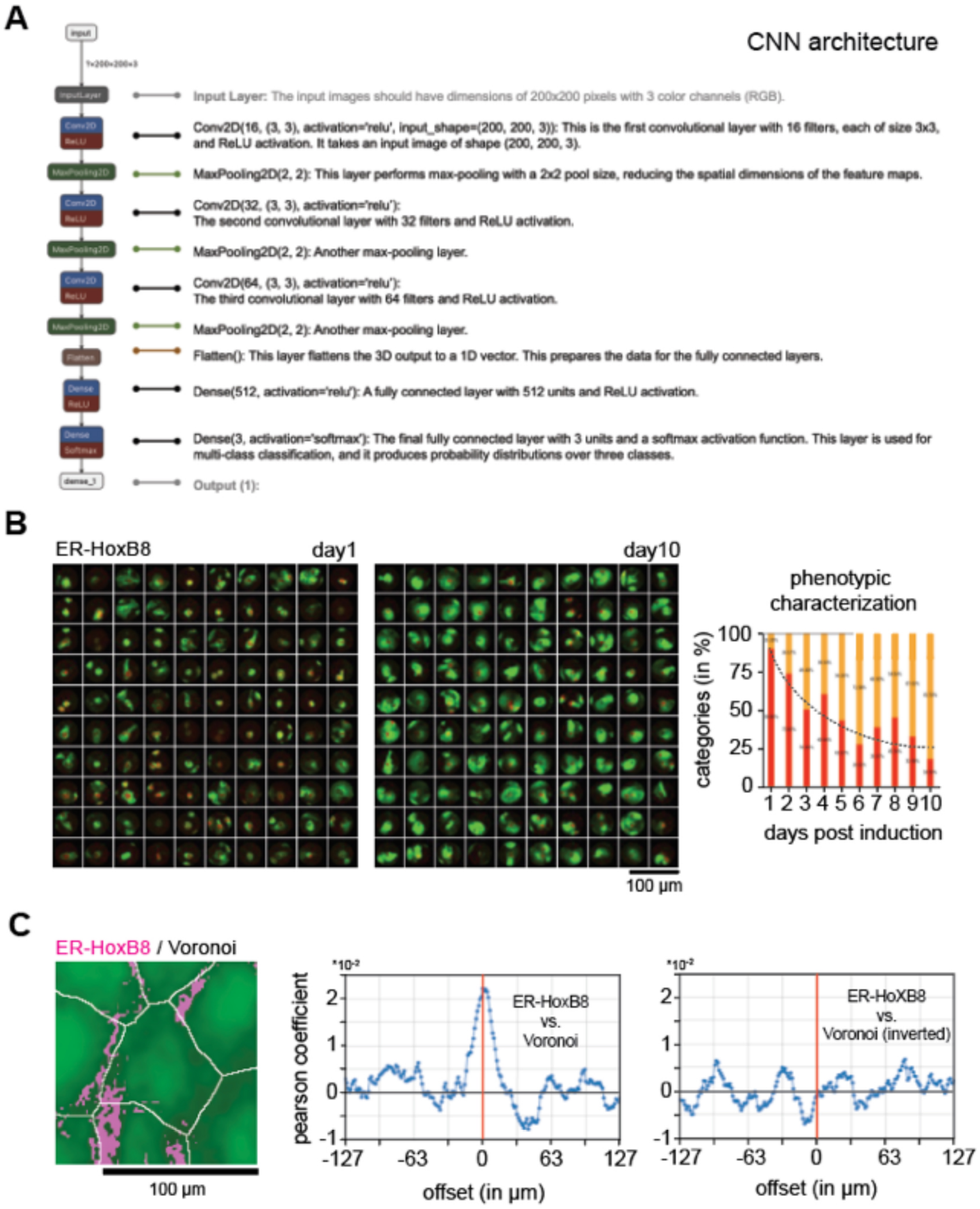

Biology Open • Supplementary information

Fig. S2. Validation of ER-HoxB8-derived monocytes/macrophages co-cultured with HUVECs. (A) Neuronal network architecture used to monitor changes in cell shape and immunostaining over time. **(B)** CNN analysis of ER-HoxB8 derived monocytes/ macrophage maturation. To the left, representative images of immature (day 1) and mature (day 10) ER-HoxB8-derived monocytes/macrophages used to train the CNN. Cells were simultaneously stained with phalloidin (green) and Hoechst (red), and all images were acquired with constant acquisition times. To the right, CNN analysis shows an increase in the number of mature ER-HoxB8-derived monocytes/macrophages (yellow) over time. Number of individual images per time point: day1=559, day2=684, day3=496, day4=161, day5=861, day6=621, day7=599, day8=690, day9=667, day10=492. **(C)** Cross-correlation analysis indicate that ER-HoxB8 derived monocyte/macrophages migrate along Voronoi. To the left, representative image of HUVECs (green) and ER-HoxB8 tracks (magenta) is shown. In the middle, cross-correlation analysis shows enrichment of cell tracks along endothelial cell-cell boundaries identified by Vornoi. As negative control, to the right, one channel was rotated by 180 degrees prior to cross-correlation analysis. Scale bars, (B, C) 100 µm.

A
Neutrophil (Panoptical Fast Staining Kit)

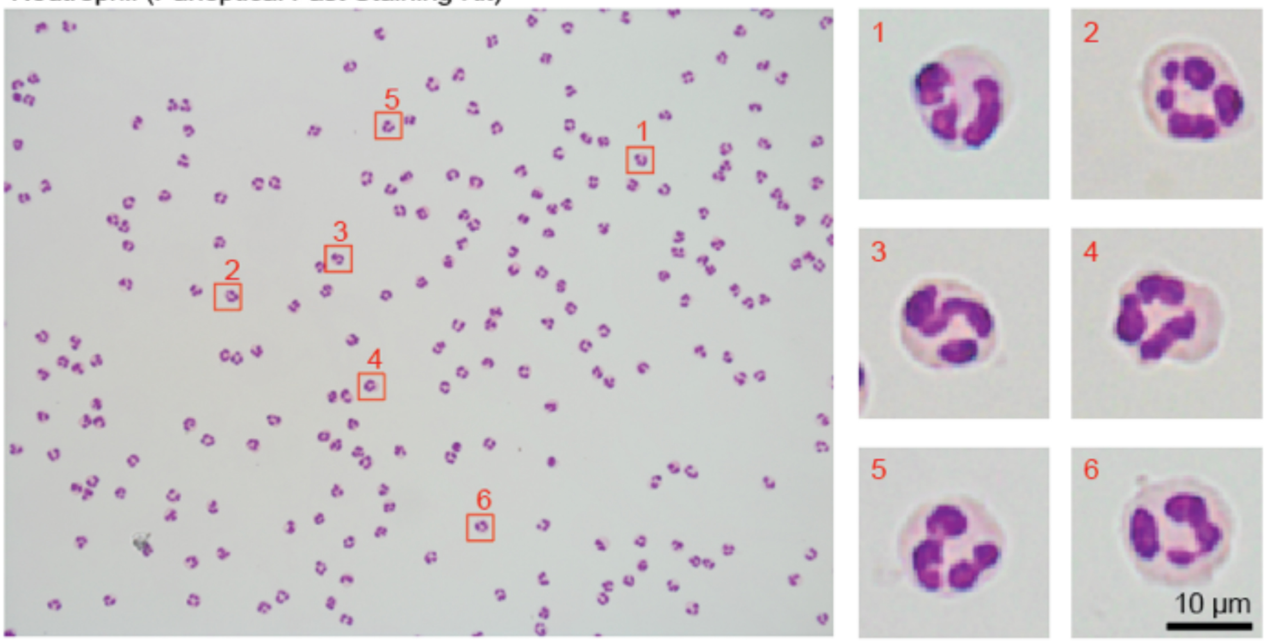

B
Neutrophil (quantification)

neutrophils	non-neutrophils	RBCs	donor sex	purity
1071	23	0	f	0,97897623
552	3	2	m	0,99102334
1006	77	0	f	0,9289012

Fig. S3. Validation of primary human neutrophils co-cultured with HUVECs. (A) Images of neutrophils upon fixation and staining with Panoptical Fast Staining Kit. **(B)** Calculation of percentage of neutrophils purity used in experiments. Scale bar, (A) 10 µm.

Biology Open • Supplementary information

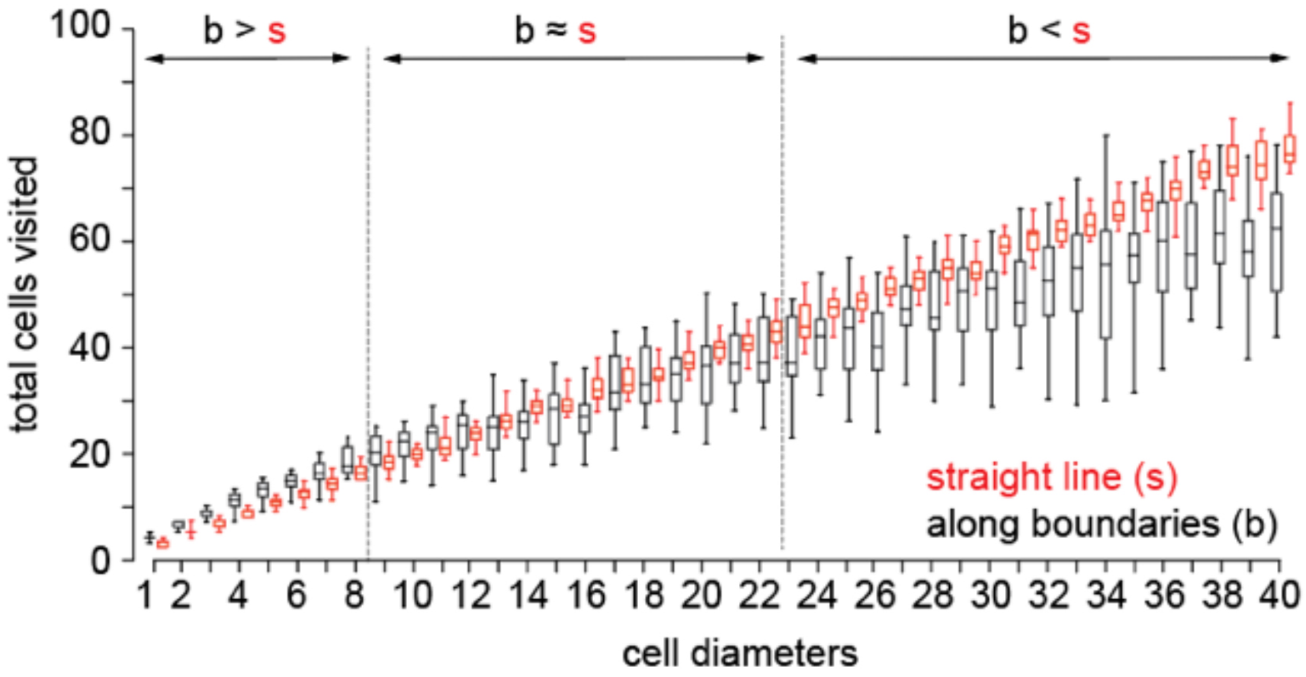

Fig. S4. Numerical model indicates that migration along cell-cell boundaries is a better search strategy for short distances, while a straight line is better for distances. Quantification of cells encountered for different walking distances is shown.

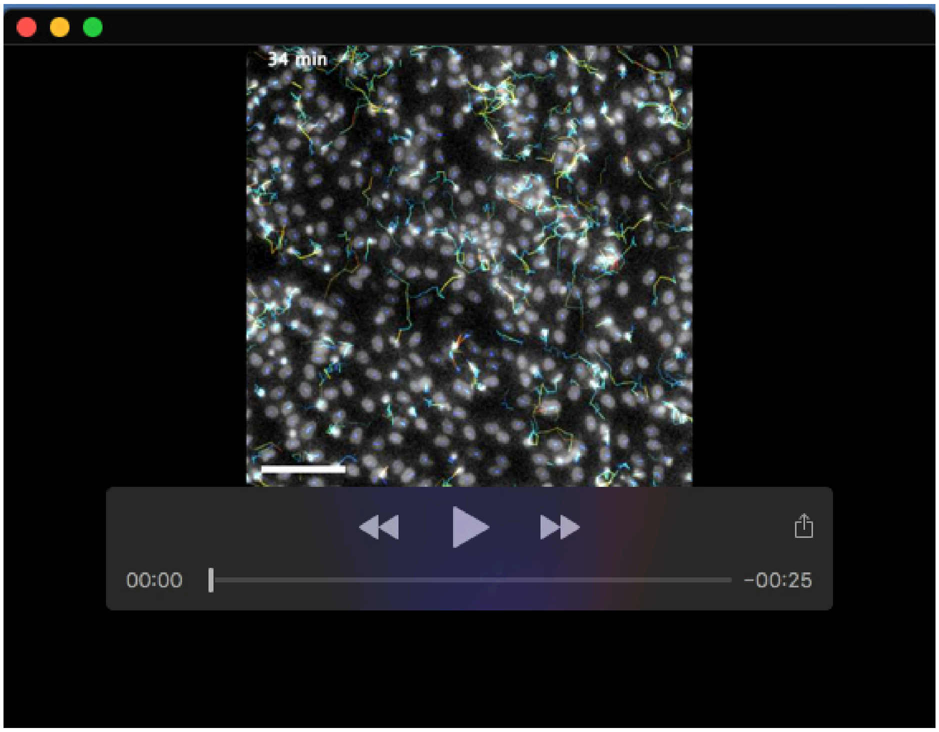

Movie 1. Overview of motion pattern of primary human monocytes on top of HUVECs (track length, 166 frames; speed, 20 fps, 1 frame per minute). Scale bar, 100 µm.

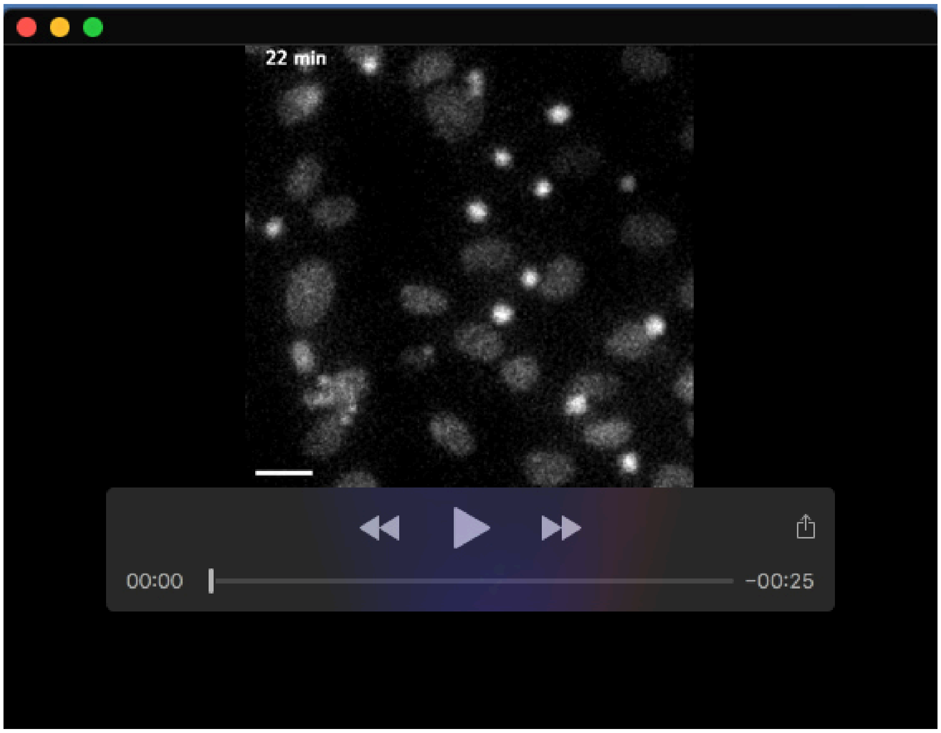

Movie 2. Motion pattern of primary human monocytes in presence of a dying cell. (track length 120 frames; 20 fps, 1 frame per minute). Scale bar, 20 µm.

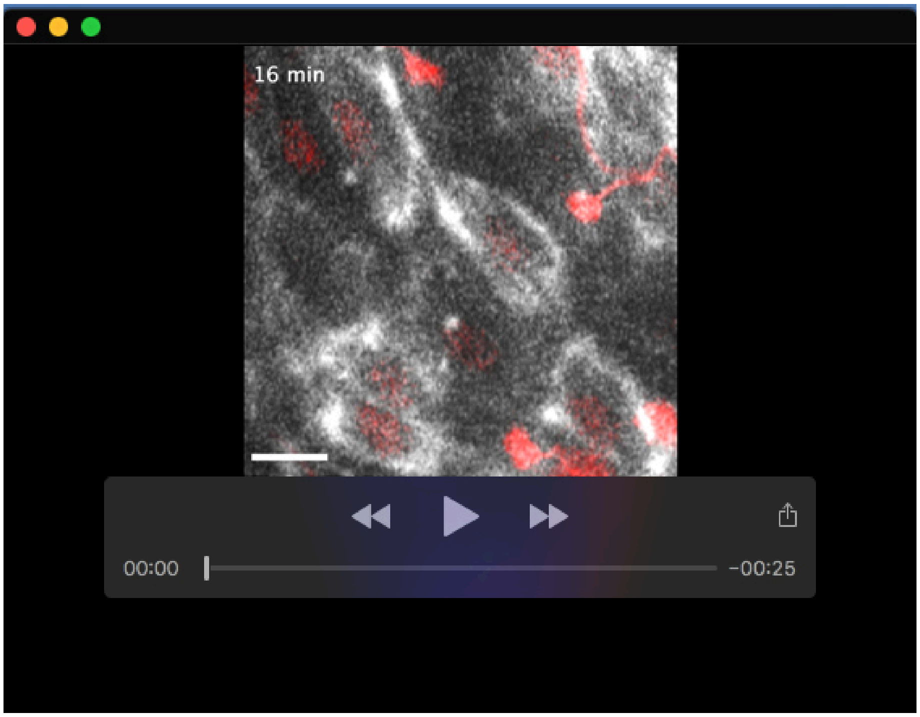

Movie 3. Motion pattern of primary human monocytes (red) on top of HUVECs (white) (track length, 75 frames; speed, 10 fps, 1 frame per minute). Scale bar, 20 µm.

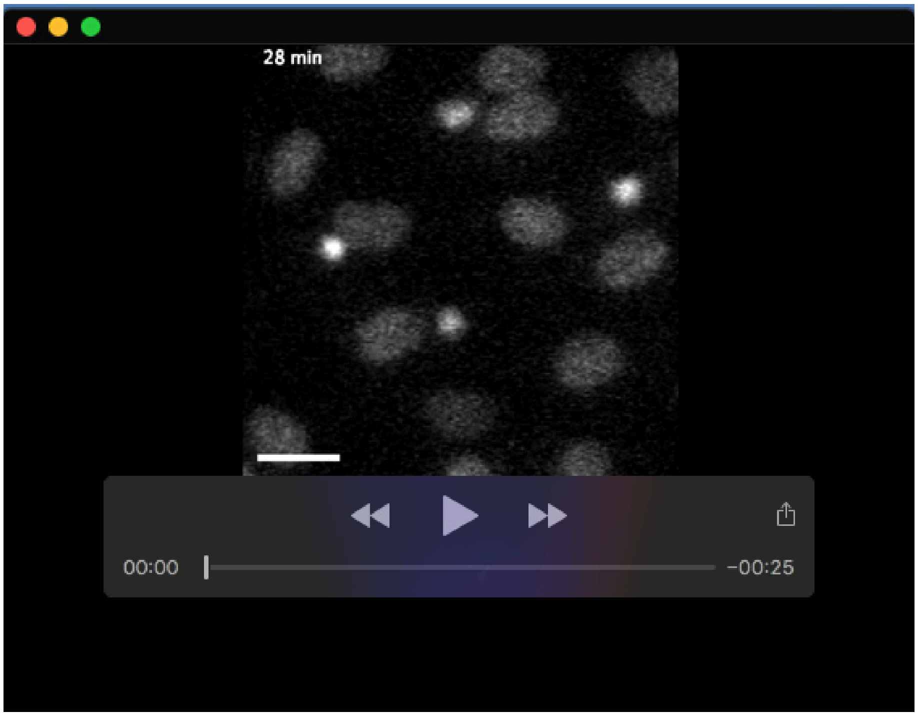

Movie 4. Motion pattern of primary human monocytes co-cultured with HUVEC (track length, 90 frames; speed, 20 fps, 1 frame per minute). Scale bar, 20 µm.

Biology Open • Supplementary information

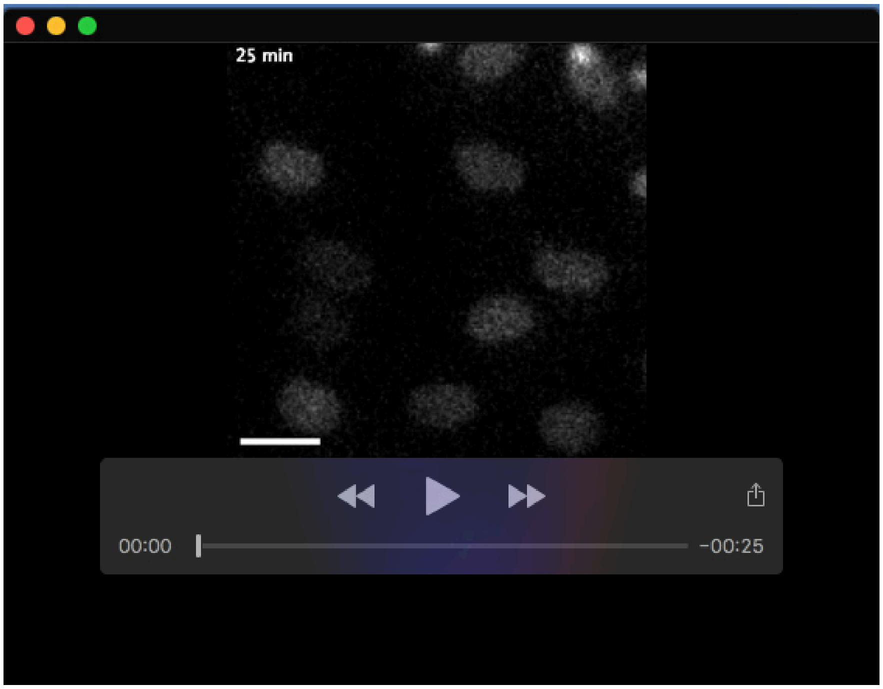

Movie 5. Motion pattern of primary human monocytes co-cultured with HUVEC (track length, 120 frames; speed, 20 fps, 1 frame per minute). Scale bar, 20 µm.

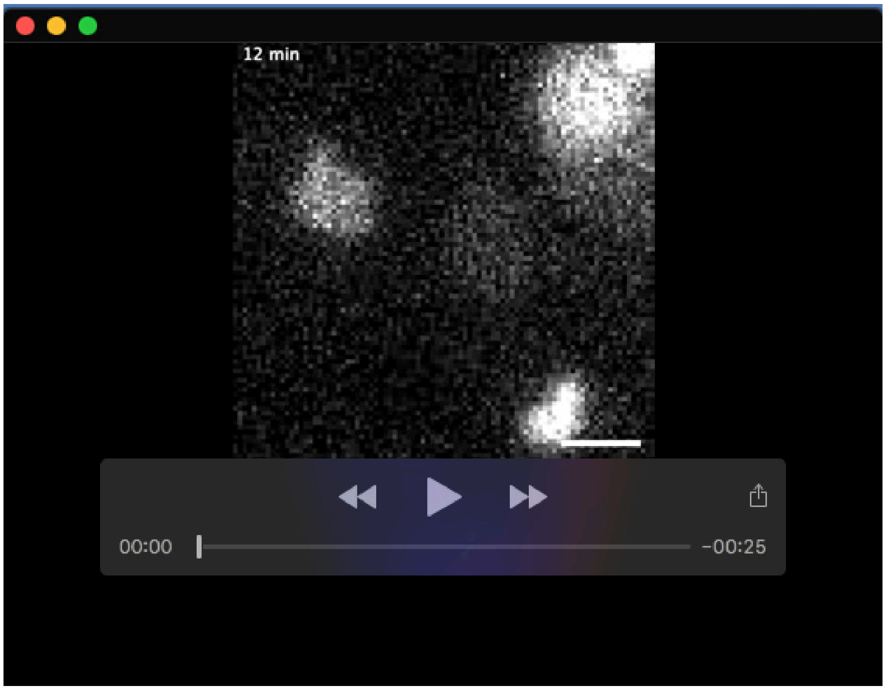

Movie 6. Transmigration of primary human monocytes through HUVEC monolayer (track length, 31 frames; speed, 10 fps, 1 frame per minute). Scale bar, 10 µm.

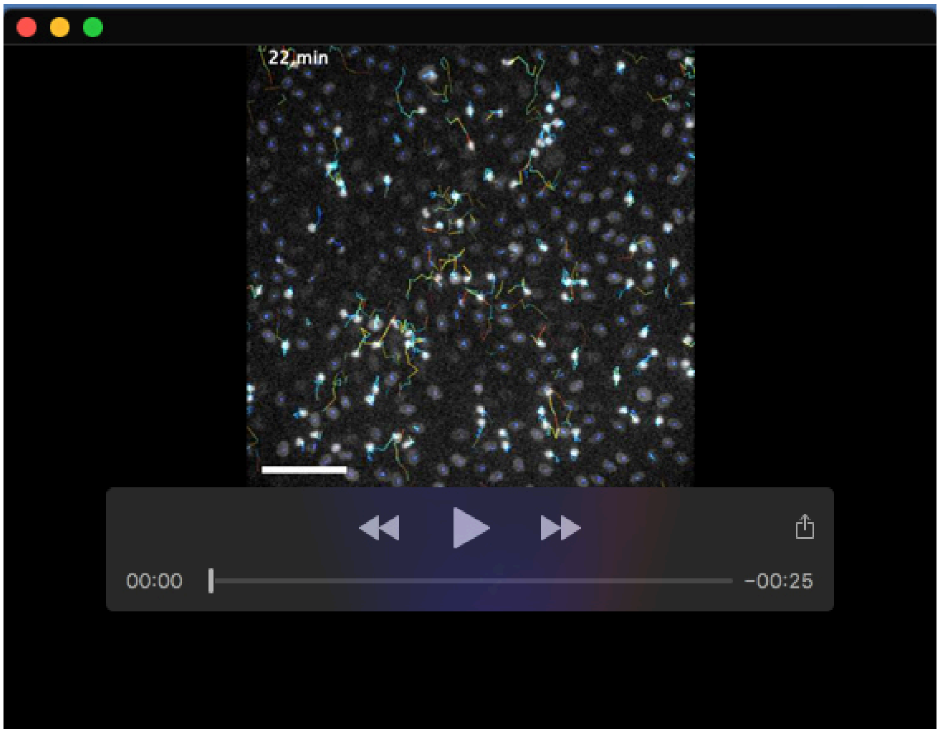

Movie 7. Motion pattern of primary human neutrophils on top of HUVECs (track length 166 frames; speed, 20 fps,2 frames per minute). Scale bar, 100 µm.

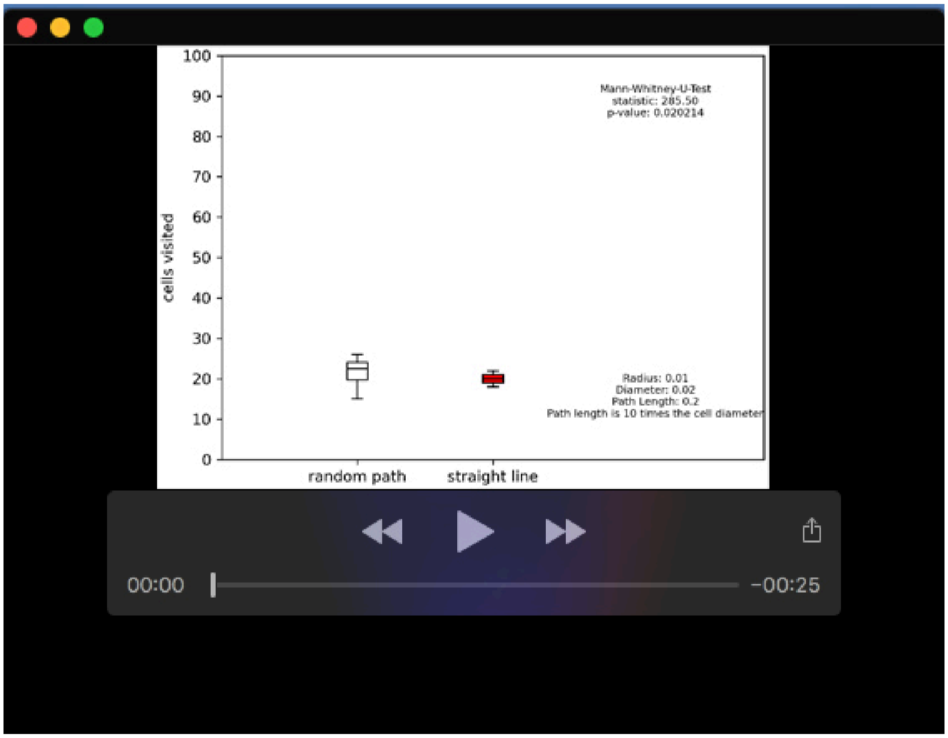

Movie 8. Numerical model depicting the number of sampled cells as function of track length for migration along cell boundaries (white, left) vs. straight line (red, right) (track length 40 frames; speed, 20 fps).
